# The Pol III transcriptome: Basic features, recurrent patterns, and emerging roles in cancer

**DOI:** 10.1002/wrna.1782

**Published:** 2023-02-08

**Authors:** Sihang Zhou, Kevin Van Bortle

**Affiliations:** 1 Department of Cell and Developmental Biology, University of Illinois Urbana-Champaign, Urbana, Illinois, USA; 2 Cancer Center at Illinois, University of Illinois Urbana-Champaign, Urbana, Illinois, USA

**Keywords:** RNA polymerase III, small RNA, noncoding RNA, cancer biology, transcription

## Abstract

The RNA polymerase III (Pol III) transcriptome is universally comprised of short, highly structured noncoding RNA (ncRNA). Through RNA–protein interactions, the Pol III transcriptome actuates functional activities ranging from nuclear gene regulation (7SK), splicing (U6, U6atac), and RNA maturation and stability (RMRP, RPPH1, Y RNA), to cytoplasmic protein targeting (7SL) and translation (tRNA, 5S rRNA). In higher eukaryotes, the Pol III transcriptome has expanded to include additional, recently evolved ncRNA species that effectively broaden the footprint of Pol III transcription to additional cellular activities. Newly evolved ncRNAs function as riboregulators of autophagy (vault), immune signaling cascades (nc886), and translation (Alu, BC200, snaR). Notably, upregulation of Pol III transcription is frequently observed in cancer, and multiple ncRNA species are linked to both cancer progression and poor survival outcomes among cancer patients. In this review, we outline the basic features and functions of the Pol III transcriptome, and the evidence for dysregulation and dysfunction for each ncRNA in cancer. When taken together, recurrent patterns emerge, ranging from shared functional motifs that include molecular scaffolding and protein sequestration, overlapping protein interactions, and immunostimulatory activities, to the biogenesis of analogous small RNA fragments and noncanonical miRNAs, augmenting the function of the Pol III transcriptome and further broadening its role in cancer.

This article is categorized under:

RNA in Disease and Development > RNA in Disease

RNA Processing > Processing of Small RNAs

RNA Interactions with Proteins and Other Molecules > Protein-RNA Interactions: Functional Implications

## 1 | INTRODUCTION

The noncoding RNA universe plays host to a wide spectrum of RNA species ranging in size from the very small, 17–25 nt microRNA (miRNAs), to large, multi-kilobase long noncoding RNAs (lncRNA) and circular RNAs (circRNAs; Cable et al., 2021). Coming in at the middle of these lower and upper bounds, RNA polymerase III (Pol III)-transcribed genes encode small RNAs between 70 and 35’ nucleotides, with subsequent post-transcriptional processing events generating shorter, mature RNAs (Watt et al., 2022). Whereas miRNAs, lncRNAs, and circRNAs generate significant attention for context-specific expression profiles and regulatory functions implicated in cancer progression, Pol III-derived transcripts are frequently considered simple housekeeping molecules integral to basic cellular processes such as translation, transcription regulation, and RNA processing. However, recent studies have illuminated a far more complex story for the Pol III transcriptome, including tissue- and cancer-specific expression profiles, novel protein interactions that connect to and modulate additional cellular processes, and post-transcriptional processing events giving rise to smaller secondary RNA fragments with distinct regulatory functions relevant to cell proliferation and cancer progression (Kessler & Maraia, 2021; Yeganeh & Hernandez, 2020).

In this review, we survey the breadth of the Pol III transcriptome by summarizing the basic features and cellular activities of Pol III-derived ncRNA in humans. The biogenesis, post-transcriptional processing, and protein interactions are explored for each ncRNA subclass, which are grouped by core functions. Beyond the central features, we consider noncanonical functions reported for each small ncRNA, ranging from molecular sequestration of specific proteins and miRNAs, activation or repression of endogenous immune signaling pathways, to the biogenesis and extracellular circulation of multifunctional small ncRNA fragments with implications for cellular microenvironments. When taken together, the Pol III transcriptome, which is commonly upregulated in cancer, is understood to overwhelmingly support and enhance cell proliferation and to promote cancer progression through mechanisms related to each ncRNA subtype. We conclude with a summary of the recurrent molecular and functional patterns that emerge as shared features of the Pol III transcriptome.

## 2 | TRANSFER RNA AND 5S RIBOSOMAL RNA: INTEGRAL COMPONENTS OF TRANSLATION

**FIGURE 1:**
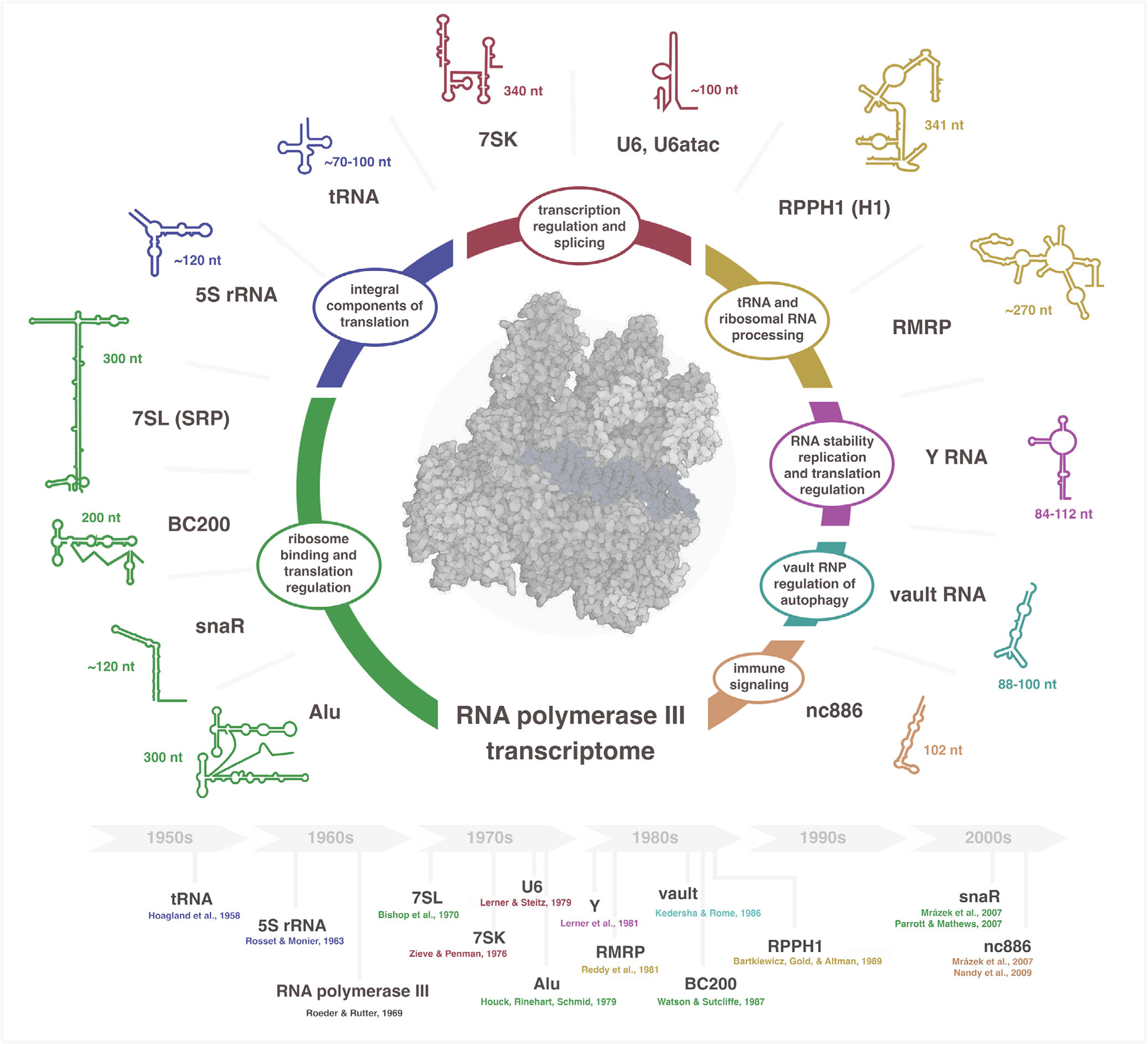
The Pol III transcriptome: small noncoding RNA with central roles in diverse cellular processes. Human Pol III-derived ncRNA subclasses are grouped by general functions, including 7SL and 7SL-derived ncRNAs involved in translation regulation (7SL, Alu, BC200, snaR), core translation ncRNAs (tRNA and 5S rRNA); small nuclear snRNAs (7SK and U6/U6atac), catalytic RNase P and RNase MRP ncRNAs (RPPH1 and RMRP), Ro60 stem-bulge ncRNAs (Y RNA), vault-associated ncRNAs (vault), and PKR regulatory ncRNA (nc886). Bottom: Historical timeline of studies related to the identification and characterization of Pol III-transcribed small ncRNA. Illustrative RNA structures were adapted from RNAcentral (7SL); (Jung et al., 2014) (BC200); RNArtist (snaR); (Chen & Yang, 2017) (Alu); (Chen, Zhang, et al., 2021) (tRNA), (Huang et al., 2020) (5S rRNA); (Eichhorn et al., 2018) (7SK); (Didychuk et al., 2018) (U6); RNAcentral (RPPH1); (Solem et al., 2015) (RMRP); (Kowalski & Krude, 2015) (Y RNA); (Mrázek et al., 2007) (vault); (Calderon & Conn, 2018) (nc886).

Discovered in 1958 as the RNA acceptor molecule of activated amino acids during protein synthesis (Hoagland et al., 1958), more than a decade before the identification of RNA polymerase III (Roeder & Rutter, 1969), tRNA and its cloverleaf secondary structure is universally known for its central role in translation (Figure 1). A few years later, 5S ribosomal RNA (rRNA) was discovered as a 5’S large subunit component in E. coli (Rosset & Monier, 1963), and is now known to be a highly conserved and integral component of the ribosome, with its three-branch secondary structure observed throughout nature (Sun & Caetano-Anollés, 2009). Here, our survey of the Pol III transcriptome begins with the features, functions, and cancer connections reported for tRNA and 5S rRNA.

### 2.1 | tRNA: Decoder of the genetic code and progenitor of multifunctional tRNAderived fragments

**FIGURE 2:**
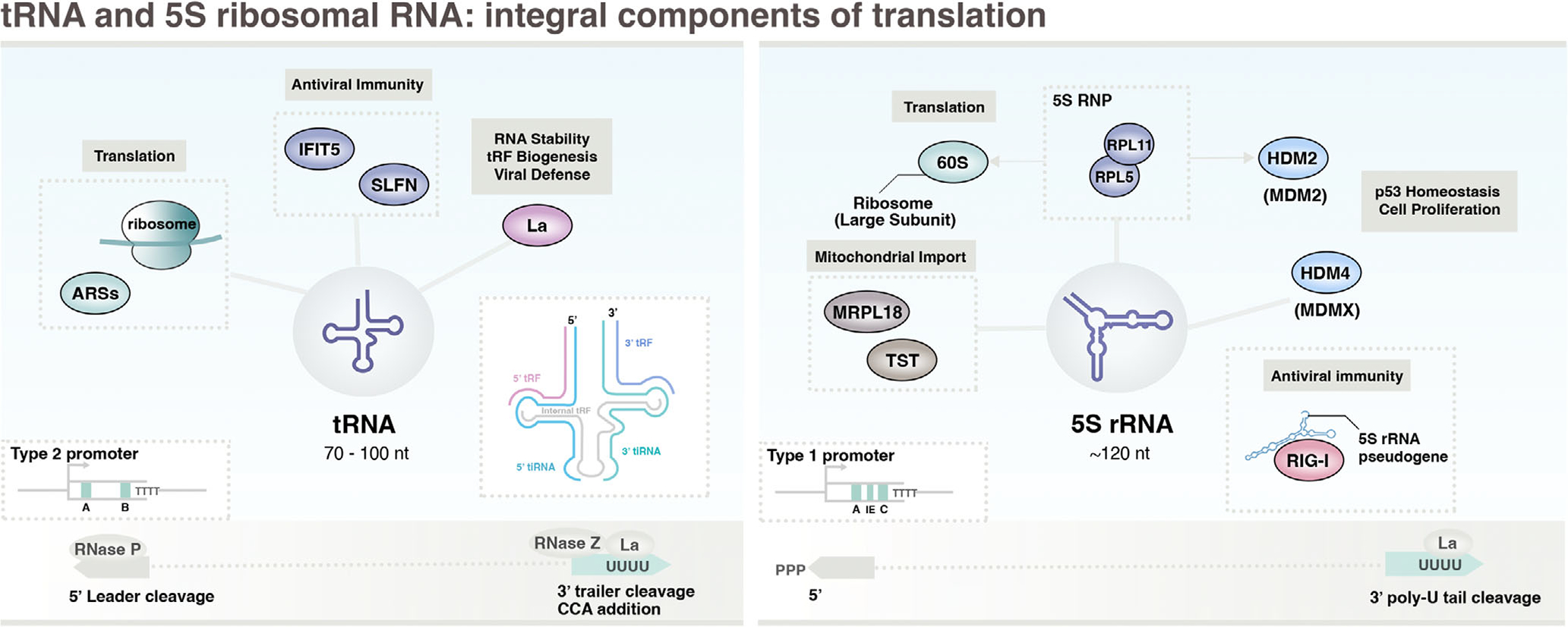
An overview of Pol III-derived tRNA and 5S rRNA. Schematic illustrates the basic gene and ncRNA features, protein interactions, and processes. RNA polymerase III is recruited to most tRNA genes via type 2 gene internal promoter sequences (A-box, B-box), whereas 5S rRNA transcription is initiated through a unique type 1 promoter architecture (A-box, C-box). Beyond the central roles of tRNA and 5S rRNA in translation, nascent tRNA and 5S are bound by chaperone protein La which, in the case of tRNA, protects tRNA from events that otherwise give rise to multifunctional tRNA fragments. Nonconical functions also include modulation of p53 homeostasis through 5S rRNA interactions with HDM2 (MDM2) and HDM4 (MDMX), and intersection with cellular immune surveillance mechanisms through interactions with pattern recognition receptor RIG-I as well as other RNA-binding proteins.

Pol III is specially tuned for transcribing small RNA genes through recruitment strategies that rely on highly conserved sequence elements which, for tRNA genes, include gene-internal A-box and B-box (“type 2”) promoter elements (Figure 2; Schramm & Hernandez, 2002). In humans, 416 high-confidence tRNA genes are currently annotated within the genomic tRNA database (GtRNAdb), including multiple copies for most tRNA isoacceptors (Chan & Lowe, 2016). Nascent, precursor tRNAs (pre-tRNAs) are bound by the Lupus autoantigen La, a protein first identified in RNPs targeted by autoimmune antibodies (also referred to as Sjogren syndrome antigen B, SSB; Yoo & Wolin, 1997). La binds to the 3’ -oligo(U) tract present on pre-tRNA and other nascent Pol III-derived transcripts, a feature driven by oligo(dT) termination signals on the non-template strand of Pol III-transcribed genes (Arimbasseri et al., 2013). La binding protects tRNA from 3’ exonuclease turnover and prevents misfolding events that can give rise to alternate secondary structures and nuclear surveillance-mediated decay (Maraia et al., 2017). Immature tRNAs engage in an array of additional protein interactions related to processing events, including RNase P (5’ cleavage), RNase Z (3’ cleavage), tRNA nucleotidyltransferases (CCA-adding enzymes), and several tRNA-modifying enzymes and proteins involved in tRNA splicing and nuclear export (Berg & Brandl, 2021). Post-transcriptional modifications of tRNA, of which there are many, influence the stability and structure of tRNA as well as translational fidelity (Tuorto et al., 2015). Following processing and maturation events, interactions with aminoacyl-tRNA synthetases (ARSs), a family of essential enzymes responsible for ligating amino acids to specific tRNAs, generate charged tRNAs available for translation (Kaiser et al., 2020).

Beyond its central role in translation, tRNAs engage in multiple noncanonical functions, many of which are actuated by tRNA-derived small RNAs (tsRNAs), small regulatory RNAs that go on to drive changes in gene expression, translation, and other biological processes. tsRNAs include tRNA halves generated by Angiogenin-mediated cleavage of tRNA anticodons, and multiple subgroups of tRNA-derived fragments (tRFs), generated by Dicer, RNase Z, and other RNase enzymes, which are classified based on biogenesis (Xie et al., 2020). As an RNA chaperone, La binding prevents interactions between tRNA and miRNA-biogenesis machinery (Hasler et al., 2016). While the 5’ cleavage of tRNA produces 5’-monophosphate ends and facilitates bypass from cytoplasmic RIG-I surveillance, tRNAs are bound by IFN-induced proteins, including IFIT5, a tetratricopeptide repeat family protein that binds both 5’-monophosphate and 5’-polyphosphate RNAs, and SLFN11/SLFN13, Schlafen family proteins with endoribonuclease activities (Yang et al., 2018). SLFN proteins cleave tRNA in response to viral infection, effectively restricting cellular tRNA and inhibiting translation.

The expression of tRNA and biogenesis of tsRNAs are dynamic processes that are regularly altered in cancer contexts with implications for disease progression. Specific tRNAs, tRNA^ARG^_CCG_ and tRNA^Glu^_UUC_, are upregulated in highly metastatic breast cancer cells and increase the translation of oncogenic transcripts bearing cognate codons, driving a proteomic shift that promotes metastatic progression (Goodarzi et al., 2016). Upregulation of initiator methionine tRNA (tRNA_i_^Met^), which alters tRNA expression profiles and increases cell proliferation (Pavon-Eternod et al., 2013), can increase the metastatic potential in melanoma skin cancer by enhancing cell migration and invasion (Birch et al., 2016). In specific hematological malignancies, altered tRNA signatures that include upregulation of valine tRNA biogenesis and changes in tRNA and tRF regulatory modifications lead to dynamic translation patterns that increase protein synthesis and can promote tumor growth (Lee et al., 2022). tRFs are reportedly capable of both pro-metastatic and tumor suppressor activities. For example, by enhancing nucleolin oligomerization, 5’-tRF^Cys^ molecules have been shown to increase the stability of specific nucleolin-bound mRNAs that encode metastasis-promoting proteins (Liu et al., 2022). In contrast, tRF^Glu^, tRF^Asp^, tRF^Gly^, and tRF^Tyr^ functionally destabilize specific mRNAs by sequestering YBX1, an RNA-binding protein that targets mRNA recognition sequences enriched within pro-oncogenic transcripts (Goodarzi et al., 2015). Deregulation and overexpression of many tRNA-interacting proteins, including chaperone La, ARSs, and tRNA-modifying enzymes such as NSUNS2, METTL1, and ELP1, are commonly observed in a multitude of cancer contexts and often implicated in both relations to aberrant tRNA dynamics as well as tRNA-independent mechanisms (Sommer & Heise, 2021; Suzuki, 2021; Zhou et al., 2020).

### 2.2 | 5S rRNA: Ubiquitous component of the large ribosomal subunit and riboregulator of p53

The smallest RNA component of the ribosome, 5S rRNA is unique among ribosomal RNAs for being derived from Pol III transcription rather than RNA polymerase I (Pol I). 5S rRNA is fundamentally important for both ribosomal assembly and function and, in humans, is encoded by repetitive arrays of canonical genes largely organized within clusters of tandem repeats on chromosome 1 (Szymanski et al., 2016; Yu & Lemos, 2018). 5S rRNA transcription is dependent on both an A-box and a unique C-box (type 1-specific) promoter element that recruits core transcription factor TFIIIA (Figure 2; Engelke et al., 1980) which, along with TFIIIC, recruit transcription factor TFIIIB prior to Pol III recruitment (Roeder, 2019). Like tRNA, nascently transcribed precursor 5S rRNA is bound by the chaperone protein La through recognition of its 3’-oligo(U) tract. Though our current understanding of human 5S rRNA processing and maturation remains limited, relevant studies in Drosophila suggest 5S rRNA undergoes 3’-exonucleolytic maturation via REXO5, a conserved 3’–’ RNA exonuclease (Gerstberger et al., 2017), generating a mature 119-nucleotide rRNA that largely retains its 5’-PPP moiety (Mori & Ichiyanagi, 2021). In contrast to tRNA, current evidence suggests 5S rRNA is largely devoid of post-transcriptional modifications (Natchiar et al., 2017; Taoka et al., 2018). In addition to canonical 5S rRNA, 5S pseudogenes are also expressed under specific conditions and, in response to viral infection, can become cytoplasmic and stoichiometrically unshielded from potential protein partners. In this context, endogenous 5S rRNA pseudogenes can activate RIG-I and downstream cytokine responses by virtue of 5’-PPP recognition (Chiang et al., 2018).

In addition to chaperone protein La, 5S rRNA interacts with ribosomal proteins RPL5 and RPL11 and together assembles a pre-ribosomal 5S RNP that is subsequently incorporated into nascent 60S ribosomes in the nucleolus. 5S rRNA also directly interacts with PICT1, RRS1, and BXDC1, factors involved in 5S RNP localization and ribosome incorporation (Sloan et al., 2013), and with TFIIIA (Pelham & Brown, 1980). 5S rRNA also interacts with mitochondrial ribosomal protein MRP-L18 and mitochondrial enzyme thiosulfate sulfurtransferase (TST), both essential for 5S rRNA import into mitochondria where 5S rRNA reportedly plays functions in mitochondrial translation as well (Smirnov et al., 2010; Smirnov et al., 2011). Beyond the core activities of the 5S RNP and ribosome in translation, 5S RNPs also independently modulate tumor suppressor protein p53 through interactions with HDM2 (MDM2), an E3 ubiquitin ligase that channels p53 to proteasome-mediated degradation. Non-ribosomal 5S RNPs sequester HDM2, resulting in p53 activation and altogether coupling downstream regulation of cell proliferation to ribosome biogenesis (Donati et al., 2013). HDM4 (MDMX), a homologous protein that inhibits p53 through direct protein interactions, requires 5S rRNA for p53 inactivation (Li & Gu, 2011), suggesting freely available 5S rRNA may contrarily inhibit p53 independently of RPL5 and RPL11.

Whether upregulation of Pol III transcription, a hallmark of cancer, contributes to cell proliferation status through a non-ribosomal 5S rRNA-HDM2/4 regulatory axis requires further investigation. However, mutation and/or misregulation of p53, HDM2, and HDM4 occur in most human cancers (Toledo & Wahl, 2006), and recent genomic studies have identified an association between 5S rDNA gene amplification with TP53 inactivation in numerous tumors (Wang & Lemos, 2017), further implicating 5S rRNA as a regulator of p53 and putative driver of p53 dysregulation in cancer. Ribosomal proteins RPL5 and RPL11, on the other hand, can inactivate oncogenic c-Myc by targeting the 3’ UTR of c-Myc mRNA, effectively downregulating c-Myc and inhibiting cell proliferation in a manner similar to p53 stabilization (Liao et al., 2014). While these results implicate the 5S RNP and 5S rRNA in c-Myc inactivation, overexpression miR-15’ and miR-383, miRNAs that target 5S rRNA, enhance c-Myc-RPL11 interactions (Wang, Ren, et al., 2015), suggesting RPL5 and RPL11 may regulate c-Myc independently of 5S rRNA. Nevertheless, the intersection between Pol III transcription and 5S rRNA with p53 homeostasis and c-Myc activity requires future research.

## 3 | 7SK AND U6 SMALL NUCLEAR RNA: TRANSCRIPTION REGULATION AND SPLICING

Most small nuclear RNA (snRNA) species are generated by Pol II transcription, however, Pol III transcribes genes encoding 7SK snRNA, the molecular scaffold of the 7SK snRNP involved in Pol II transcription regulation, and U6 and U6atac snRNA, components of splicing machinery retained in the nucleus. 7SK snRNA was first described as a predominantly cytoplasmic small RNA in HeLa cells (Zieve & Penman, 1976; Figure 1), though it is now recognized for its conserved role in RNA polymerase II transcription regulation via inhibition of the positive transcription elongation factor b (P-TEFb) complex in the nucleus (Nguyen et al., 2001; Yang et al., 2001). U6 snRNA is a core component of the major spliceosome first discovered in immune precipitates with other uridine-rich snRNAs (Lerner & Steitz, 1979), and U6atac snRNA similarly functions as a paralogous RNA component of the minor spliceosome (Tarn & Steitz, 1996). Here we explore the basic features of both classes of Pol III-derived snRNAs and recent evidence linking 7SK and U6 snRNA dysregulation to cancer.

### 3.1 | 7SK snRNA: Dynamic regulatory switch for P-TEFb sequestration and transcription inhibition

**FIGURE 3:**
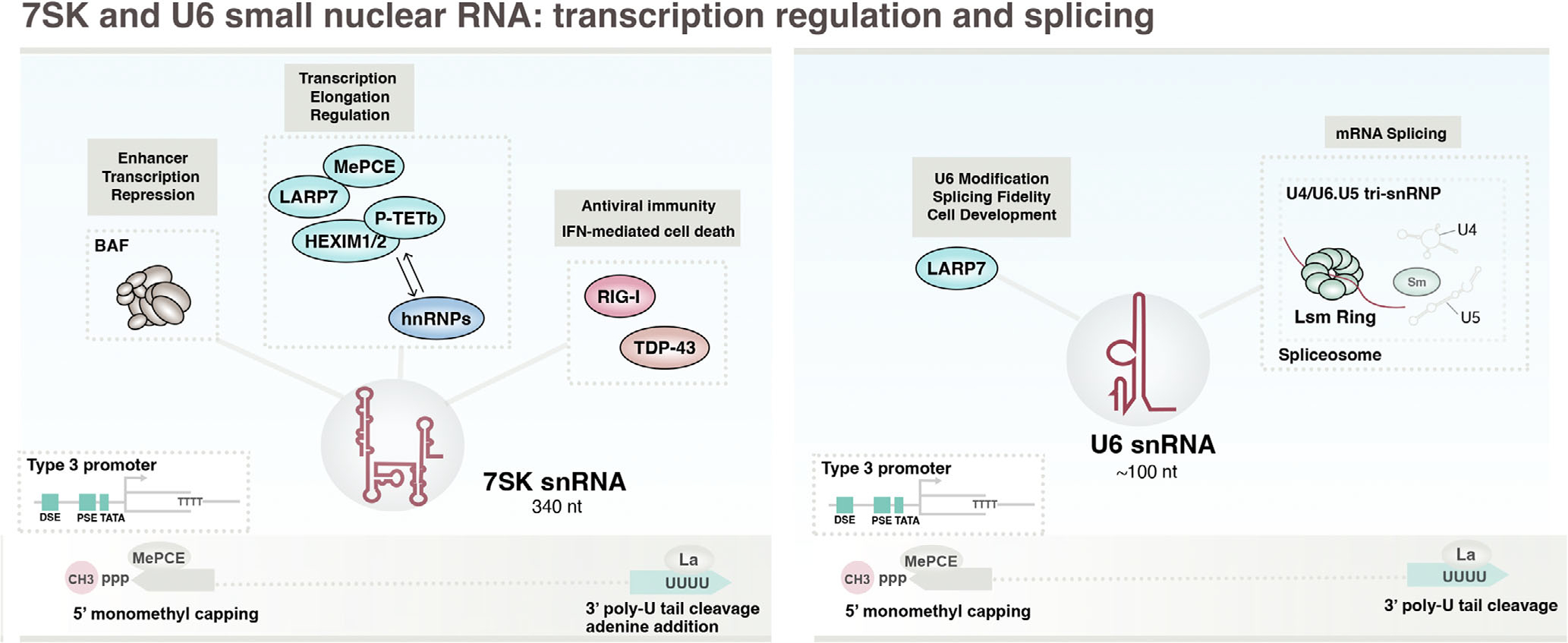
Pol III-transcribed 7SK and U6 are small nuclear (sn)RNAs with key roles in transcription regulation and splicing, respectively. Both 7SK and U6 snRNA genes feature type 3 promoter architectures, which depend on upstream regulatory sequences rather than gene-internal elements. 7SK and U6 undergo 5’ modifications important for snRNA stability and shielding of immunostimulatory 5’-PPP detection. Through RNP formation with P-TEFb and other proteins, 7SK functions as a general repressor of Pol II transcription at both paused genes and, through interactions with the BAF chromatin remodeler, enhancers. U6 and U6atac snRNA are integral and catalytic components of the major and minor spliceosomes, respectively.

7SK is derived from a single constitutively active Pol III-transcribed gene possessing a “type 3” promoter architecture that relies on proximal and distal upstream regulatory elements instead of gene internal elements (Figure 3; Murphy et al., 1986). Nascent precursor 7SK is bound by La through its 3’-oligo(U) tract, which subsequently undergoes terminal uridine removal of between 1 and 3 nucleotides followed by 3’-adenylation (Sinha et al., 1998). Processing of pre7SK also includes addition of a 5’ monomethyl cap (5’-CH3-PPP), catalyzed by methylphosphate capping enzyme (MePCE; Yang et al., 2019), which is important for 7SK stability and interactions with P-TEFb (Jeronimo et al., 2007). 7SK snRNA is approximately 340 nucleotides in length and folds into a secondary structure that features four major hairpin domains that mediate interactions with 7SK snRNP proteins (Wassarman & Steitz, 1991).

The highly abundant Pol III-derived 7SK snRNA engages in a sophisticated, dynamic network of protein interactions central to its role in sequestering P-TEFb. The core 7SK snRNP complex is comprised of LARP7, a La-related protein that similarly interacts with 3’-uridines in 7SK snRNA (Bayfield et al., 2010), and MePCE, which remains bound following 5’-monomethylation of 7SK. The binding of both LARP7 and MePCE induces a conformational switch in 7SK essential for downstream interactions and function (Yang et al., 2022). The constitutive MePCE-7SK-LARP7 snRNP recruits HEXIM1/2, paralogous proteins that are induced by stress and nucleotide depletion (Tan et al., 2016) and which form protein dimers that bind the 5’-hairpin structure of 7SK snRNA. HEXIM in turn, through a conformational change induced by 7SK snRNA binding, binds to P-TEFb and specifically inactivates Cdk9 (Li et al., 2005), functionally preventing P-TEFb from facilitating promoter-proximal pause release and Pol II transcription elongation (Bugai et al., 2019). Chromatin-associated 7SK also interacts with the BAF chromatin remodeling complex and inhibits transcription at specific enhancers (Flynn et al., 2016).

The P-TEFb-bound 7SK snRNP itself is reversibly regulated through stress and growth signaling pathways, which can facilitate the release and re-activation of P-TEFb (Olson et al., 2022). Proteolytic cleavage of MePCE by Jumonji C-domain-containing protein 6 (JMJD6), for example, can release P-TEFb from the 7SK snRNP (Lee et al., 2020). JMJD6 can also demethylate 7SK through long-range enhancer–promoter interactions, ensuring its degradation and local activation of P-TEFb at specific promoters (Liu et al., 2013). The core 7SK snRNP can also assemble into an alternate complex through interactions with RNA helicase A and heterogeneous nuclear ribonucleoproteins (hnRNPs) A1, A2/B1, R, and Q, effectively controlling the availability of 7SK for P-TEFb sequestration (van Herreweghe et al., 2007). Beyond the 7SK snRNP, 7SK snRNA is also bound by TDP-43, a nuclear RNA-binding protein that influences the expression, subcellular localization, and turn-over of multiple Pol III-derived ncRNA. Depletion of TDP-43 induces cytoplasmic accumulation of 7SK snRNA and RIG-I-dependent immune signaling events, suggesting levels of immunostimulatory 5’-PPP 7SK are limited by TDP-43 function (Dunker et al., 2021).

As a generally repressive regulator of transcription, both 7SK and the 7SK-snRNP have been described as putative tumor suppressors: loss of LARP7 and the 7SK snRNP leads to upregulation of epithelial-mesenchymal transition (EMT) associated genes and increased invasion and metastasis in breast cancer (Ji et al., 2014), and exogenous over-expression of 7SK inhibits cell proliferation in cancer cell lines (Keramati et al., 2015). 7SK snRNA expression levels are also commonly decreased in a multitude of cancer contexts, including breast, colon, and cancer cell lines (Abasi et al., 2016), and surveys of 7SK snRNA expression in tongue squamous cell carcinoma (TSCC) recently identified negative correlations between 7SK levels and tumor size across 73 TSCC patients (Zhang et al., 2021). Downregulation of LARP7, which reduces the level of 7SK, has also been linked with gastric cancers (Cheng et al., 2012). While these reports indicate that 7SK may serve as a potential biomarker and therapeutic cancer target, recently uncovered connections between 7SK and BAF also raise important questions regarding the involvement of 7SK in cancers characterized by BAF mutations, which are collectively present in more than 20% of human cancers (Hodges et al., 2016). Likewise, JMJD6 is also frequently altered in cancers with high expression and poor outcomes reported across many cancer contexts (Wang et al., 2022), potentially through mechanisms that include dysregulation of 7SK snRNP function.

### 3.2 | U6 and U6atac snRNA: Uridine-rich major and minor spliceosomal small nuclear RNAs

Human U6 snRNA is generated from multiple genes of varying transcriptional activity, with most featuring type 3 promoter architectures as described for 7SK (Figure 3; Kunkel & Pederson, 1988). U6 snRNA processing includes 5’ monomethyl cap addition by MePCE (Jeronimo et al., 2007; Shimba & Reddy, 1994), and 3’-end processing events that include oligouridylation by TUT1, a terminal uridylyl transferase (Trippe et al., 2006), followed by 3’ trimming by exonuclease USB1 (Nomura et al., 2018). U6 also undergoes post-transcriptional modification at internal positions, including pseudouridylation, 2’-O’methylation, and m^6^A and m^2^G methylation at specific residues (Didychuk et al., 2018). U6 is approximately 100 nucleotides, whereas human U6atac is a slightly larger 120-nucleotide snRNA and features an extended 3’-tail and additional stem-loop structure necessary for the formation of the minor spliceosome (Dietrich et al., 2009). The analogous 5’ monomethyl cap of human U6atac snRNA is bound by additional proteins, RBM48 and ARMC7, which help stabilize the conformation of the catalytic center of the minor spliceosome (Bai et al., 2021).

The major and minor spliceosomes are dynamic, macromolecular complexes made up of multiple snRNPs that are sub-assembled via uridine-rich snRNAs and associated proteins (Wilkinson et al., 2020). U6 and U6atac snRNAs generated by Pol III transcription play integral roles in coordinating splicing catalysis of precursor messenger RNA (pre-mRNA) by the major and minor spliceosomes, respectively. The major spliceosome, comprised of U1, U2, U4, U5, and U6 snRNPs and additional proteins, is dynamically formed on canonical introns (Hoskins et al., 2011). During the splicing cycle, U6 snRNA establishes a triple snRNP subcomplex through stable base pairing with Pol II-derived U4 snRNA and through interactions with the U5 snRNP (U4/U6.U5 tri-snRNP; Wan et al., 2016). Pre-assembled U4/U6.U5 snRNPs go on to interact with pre-spliceosomal complexes assembled on introns by U1 and U2 snRNPs, wherein U1 and U2 snRNAs interact and recognize 5’ splice site and branch sites, respectively. Activation of the assembled spliceosome involves rearrangement and ejection of U1 and U4 snRNPs, leaving behind a U2/U6.U5 complex that interacts with additional proteins to catalyze splicing (Matlin et al., 2005). In a similar fashion, U6atac catalyzes the chemical splicing reaction of the minor spliceosome, which recognizes noncanonical splice sites and is comprised of U11, U12, U4atac, U5, and U6atac snRNPs (Turunen et al., 2013). The protein components of most major and minor spliceosomal snRNPs include an overlapping set of seven Sm RNA-binding proteins, which together form a heteroheptameric ring-shaped complex. However, both U6 and U6atac snRNAs are instead uniquely bound by a heptamer of Sm-like (Lsm) proteins (Zhou et al., 2014), as well as SART3, a nuclear RNA-binding protein and spliceosome recycling factor, all of which are thought to function as RNA chaperones for U6 and U4 snRNP reassociation following splicing (Jandrositz & Guthrie, 1995).

U6 snRNA, often tested as a potential reference gene in miRNA studies, is consistently upregulated in a variety of cancer contexts, suggesting U6 snRNA may instead serve as a potential diagnostic marker (Lou et al., 2015). U6 snRNA levels are reportedly elevated in tumors derived from breast, colon, kidney, liver, lung, and ovary carcinomas relative to normal tissue, and elevated U6 snRNA levels are detected in plasma from patients with breast cancer (Záveský et al., 2022). While multiple mechanisms may underlie increased U6 snRNA levels in cancer, the consequence of U6 overexpression is likely to influence splicing, which is commonly dysregulated in cancer (Oltean & Bates, 2014). In this regard, mutations in spliceosomal subunits or events that otherwise disrupt the balance of snRNAs and associated RNA-binding proteins lead to global changes in splicing, effectively altering the landscape of proto-oncogene and tumor suppressor isoforms that can give rise to oncogenesis (El Marabti & Younis, 2018). Similarly, targeted disruption of U6 snRNA interactions and stability by miR-10b, an oncogenic miRNA that promotes metastasis (Sheedy & Medarova, 2018), perturbs global splicing in glioblastoma, resulting in isoform changes that influence cell viability in malignant glioma (El Fatimy et al., 2022). Overexpression of Lsm proteins is also observed in multiple cancer types (Long et al., 2014; Zhuang et al., 2022), and nearly all U6 snRNP LSMs are associated with poor prognostic outcomes among breast cancer patients (Ta et al., 2021). SART3 overexpression is also an unfavorable prognostic indicator in multiple cancers, including melanoma (Timani et al., 2018), altogether linking both U6 and its network of protein interactions to dysfunction in cancer and as predictors of clinical outcomes.

## 4 | RPPH1 AND RMRP: RNA COMPONENTS OF RIBONUCLEASE P/MRP FAMILY RNPS

Pol III-transcribed RPPH1 (H1) ncRNA is the catalytic RNA component of RNase P, the site-specific endonuclease responsible for the cleavage of pre-tRNA 5’-leader sequences previously discussed (Bartkiewicz et al., 1989). The closely related eukaryotic ribozyme, RNase MRP, similarly recognizes and cleaves pre-rRNA through a process that involves Pol III-transcribed RMRP ncRNA, first discovered as a small nuclear RNA present in the nucleolus (Figure 1; Reddy et al., 1981). Most RNase P and RNase MRP proteins are shared, and the two complexes have been shown to exist at approximately equal levels in lower eukaryotes (Esakova & Krasilnikov, 2010). Here we explore the biogenesis, functions, and cancer connections for both RPPH1 and RMRP.

### 4.1 | RPPH1: Catalytic RNA component of RNase P: From tRNA recognition and 5’ cleavage to transcription regulation and miRNA sequestration

**FIGURE 4:**
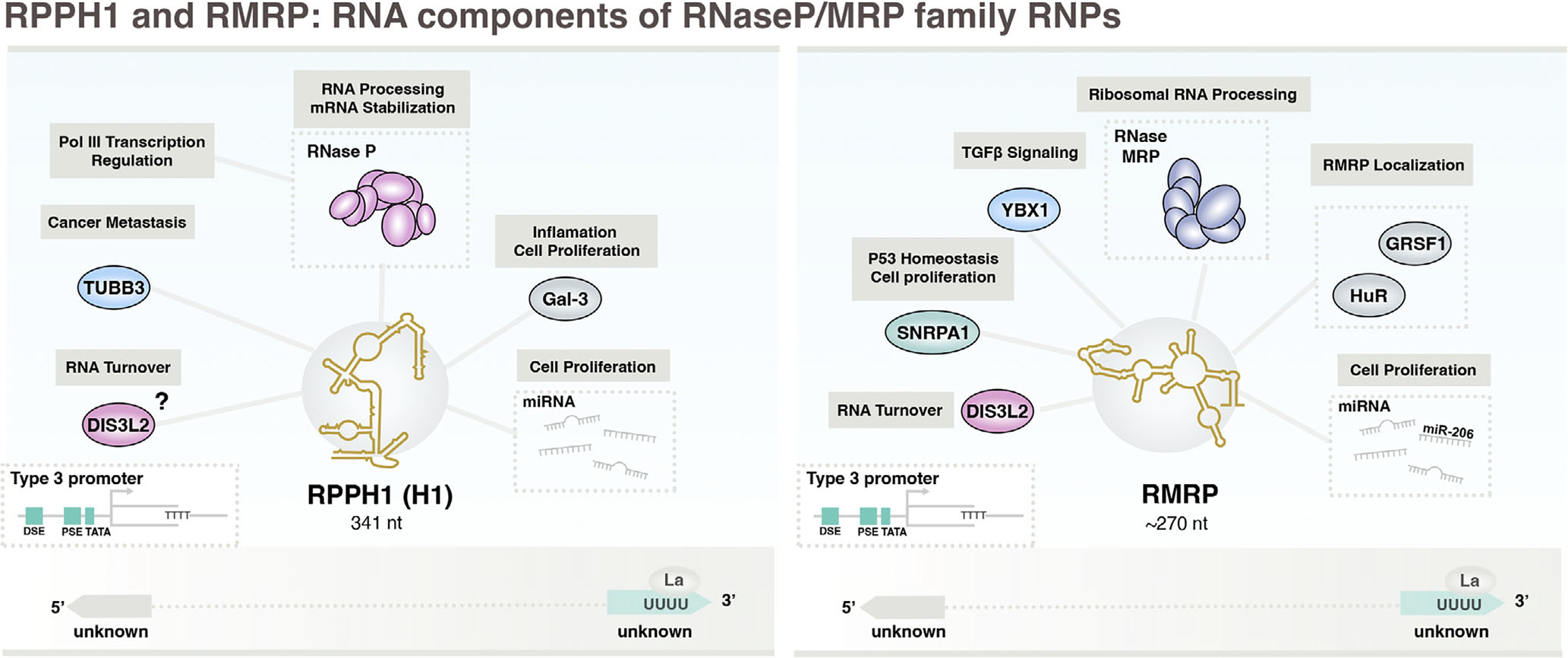
An overview of RPPH1 (H1) and RMRP, two highly related catalytic ncRNAs involved in tRNA and ribosomal RNA processing, respectively. RPPH1 and RMRP genes feature type 3 promoter architectures, reliant on upstream sequence elements. RPPH1 is the catalytic RNA component of RNase P, which recognizes and cleaves the 5’ leader sequence of nascent tRNA. RMRP mediates endonucleolytic cleavage of pre-rRNA by RNase MRP. Both RPPH1 and RMRP sequester specific miRNAs with downstream consequences on cell growth and proliferation. Additional noncanonical functions include transcription regulation (RPPH1/RNase P) as well as interactions with proteins that intersect growth signaling pathways. Cytoplasmic RMRP undergoes 3’-oligouridylation and is channeled into DIS3L2-mediated decay. The function of similarly identified DIS3L2-RPPH1 interactions, however, remain less clear.

RPPH1 (also known as H1 ncRNA) is 341 nucleotides in length and generated from a single gene with type 3 upstream promoter elements (Figure 4; Hannon et al., 1991). The *RPPH1* gene is also occupied by RNA polymerase II (Pol II) and sensitive to Pol II inhibition, suggesting both Pol II and Pol III contribute to the biogenesis of RPHH1 ncRNA (James Faresse et al., 2012). While comparatively less is currently understood about the processing events of RPPH1, including 5’ and 3’ events, global analysis of m^5^C sites identified NSUN2-dependent modifications of RPPH1 ncRNA (Squires et al., 2012). RPPH1 secondary structure features two independently folding and conserved domains, the specificity S-domain involved in pre-tRNA substrate recognition, and the catalytic C-domain containing the active site for tRNA cleavage (Perederina & Krasilnikov, 2010).

RPPH1 ncRNA is bound by an ensemble of protein subunits, including RPP14, RPP20 RPP21, RPP25, RPP29, RPP3’, RPP38, RPP40, hPOP1, and hPOP5 (Jarrous & Reiner, 2007) which functionally stabilize RPPH1 for pre-tRNA substrate recognition and cleavage (Wu et al., 2018). The RNase P ribozyme is a large ~45’ kDa complex and, beyond its highly conserved role in tRNA processing, reportedly functions in transcription regulation, as depletion of RNase P results in significant loss of Pol III transcription activity (Reiner et al., 2006; Reiner et al., 2008). RNase P is found in coimmunoprecipitates with core Pol III proteins and co-localizes to Pol III-transcribed genes by chromatin immunoprecipitation (ChIP). The co-localization and positive regulation of Pol III transcription by RPPH1 and RNase P may indicate a potential coordinated feedback mechanism, connecting protein synthesis in the form of RNase P assembly with the biogenesis of nascent RPPH1, tRNA, and other Pol III-derived small ncRNA (Jarrous et al., 2022).

Independently of RNase P, RPPH1 ncRNA interacts with additional proteins as well as specific miRNAs to functionally promote cell growth and proliferation, and growing evidence suggests that these mechanisms drive tumorigenesis and metastasis in a variety of cancers. RPPH1 is upregulated and associated with poor survival outcomes in patients with gastric cancer (Yue et al., 2020), colorectal cancer (Liang et al., 2019), and acute myeloid leukemia (AML; Lei et al., 2019). In colorectal cancer (CRC) tissues, RPPH1 induces epithelial-mesenchymal transition (EMT) by preventing ubiquitination of β-tubulin TUBB3 and promotes local macrophage polarization through exosomal transfer of CRC-expressed RPPH1 (Liang et al., 2019). RPPH1 also enhances cell proliferation in breast cancer and AML cell lines by mechanistically antagonizing specific miRNAs, suggesting RPPH1 may function as a molecular miRNA sponge (Lei et al., 2019; Zhang & Tang, 2017). Consistent with and in addition to these reports, a novel circular form of RPPH1, circ-RPPH1, is elevated in breast cancer, cervical cancer, and hepatocellular carcinoma and in all cases promotes proliferation and tumor growth by sequestering specific miRNAs (Li et al., 2022). The canonical form of RPPH1 ncRNA also interacts with Galectin-3 (Gal-3) and activates related signaling pathways that drive cell proliferation in mesangial cells (Zhang et al., 2019). Taken together, RPPH1 appears likely to play an important role in coupling Pol III transcription to cell growth mechanisms and holds future promise as a potential biomarker and target in cancer contexts.

### 4.2 | RMRP: Catalytic RNA component of RNase MRP and molecular miRNA sponge

RNase MRP is a eukaryote-specific ribonucleoprotein complex that is structurally and evolutionarily related to RNase P. The RNase MRP complex catalyzes endonucleolytic cleavage of pre-ribosomal RNA in the nucleolus (Chang & Clayton, 1987; Kiss & Filipowicz, 1992). The RNA component of MRP (RMRP), first identified in RNP immunoprecipitates as nucleolar RNA 7–2, is similar in many respects to RPPH1, beginning with an analogous type 3 gene promoter architecture and a relatively large Pol III-derived length of ~270 nucleotides (Figure 4; Yuan & Reddy, 1991). Like RPPH1, the secondary structure of RMRP includes a highly similar catalytic C-domain from which multiple stem domain structures extend. However, RMRP diverges from RPPH1 in its specificity S-domain, which underlies the remodeling of the RNase MRP substrate binding pocket to confer distinct specificity for rRNA (Perederina et al., 2020). While the post-transcriptional processing and modification events of RMRP ncRNA remain largely unclear, m^6^A methylation enhances RMRP stability (Yin et al., 2021). Through interactions with a shared subset of RNase P proteins, human RNase MRP mediates endonucleolytic cleavage at a specific site along internal transcribed spacer 1 (ITS1) of pre-rRNA. MRP is also capable of cleavage on multiple tested fragments spanning precursor rRNA, including fragment sequences that otherwise increase in cells lacking MRP RNA, suggesting RNase MRP may catalyze the cleavage of pre-rRNA at multiple sites in humans (Goldfarb & Cech, 2017). In line with this finding, RNase MRP recognizes short, loosely defined consensus sequences in S. cerevisiae, suggesting RNase MRP-mediated cleavage events may extend beyond our current understanding (Lan et al., 2020).

Though primarily localized to the nucleus, nuclear export of RMRP can occur through interactions with RNA-binding protein HuR and association with Exportin 1 (XPO1). Mitochondrially imported RMRP is retained through interactions with an additional RNA-binding protein, GRSF1, ostensibly important for RMRP-mediated mitochondrial DNA replication priming (Noh et al., 2016). Non-nuclear RMRP is also highly enriched in pulldown experiments of a catalytically inactive form of DIS3L2, a 3’–5’ exonuclease involved in surveillance and turnover of highly structured aberrant transcripts (Pirouz et al., 2016). RMRP ncRNA, which accumulates in the cytoplasm in cells lacking DIS3L2, undergoes extensive 3’ oligouridylation, suggesting cytoplasmic RMRP is normally channeled for DIS3L2-mediated decay (DMD) by terminal uridylyl transferase activity (TUTase). Interestingly, RPPH1 is similarly bound by DIS3L2, but does not increase in uridylation, indicating TUTase-independent turnover of RPPH1, or a unique function of RPPH1-DIS3L2 interaction (Pirouz et al., 2016). RMRP is also detected in extracellular vesicles and shown to promote local cell viability through exosomal transfer, suggesting RMRP that escapes both nuclear localization and cytoplasmic DMD may ultimately drive growth within a cellular microenvironment (Hewson et al., 2016).

Like RPPH1, RMRP ncRNA is similarly overexpressed and associated with unfavorable prognoses across a multitude of cancer contexts, and evidence suggests RMRP also contributes to proliferation through novel protein interactions and miRNA sequestration. RMRP is upregulated in non-small cell lung cancer (NSCLC) and promotes proliferation, invasion, and migration by interacting and recruiting YBX1 to the TGF-β receptor 1 (TGFBR1) promoter, upregulating TGFBR1 and enhancing the TGFBR1/SMAD2/SMAD3 growth signaling pathway (Yin et al., 2021). RMRP is also upregulated in colorectal cancer, where RMRP has been shown to inactivate tumor suppressor p53 through a mechanism that involves nuclear sequestration of SNRPA1 and enhanced p53 turnover (Chen, Hao, et al., 2021). Analogous to RPPH1, RMRP is also implicated as a molecular sponge for specific miRNAs that otherwise target genes associated with growth, including miR-206, in which case RMRP overexpression neutralizes miR-206 levels and activities in lung adenocarcinoma (Meng et al., 2016), neuroblastoma (Pan et al., 2019), bladder cancer (Cao et al., 2019), and hepatocellular carcinoma contexts (Hongfeng et al., 2020). RMRP is reported to counteract the activities of numerous additional miRNAs, in all cases promoting cell proliferation, suggesting RMRP may also serve as a promising target for anti-cancer strategies.

## 5 | 7SL AND ITS EVOLUTIONARY DERIVATIVES: TRANSLATION REGULATION AND BEYOND

The signal recognition particle (SRP) RNA, 7SL, functions as a core component of the SRP RNP, which recognizes and directs presecretory proteins to translocation machinery at the ER membrane (Segev, 2009). In humans, 7SL is also the evolutionary ancestor to 7SL-derived short interspersed nuclear elements (SINEs), which include primate Alu elements dispersed at high density throughout the human genome (Ullu & Tschudi, 1984). Additional Pol III-transcribed genes have, in turn, been exapted from specific Alu sequences. *BCYRN1*, for example, is evolutionarily derived from an Alu monomer sequence that underwent a series of substitution and insertion events (Kuryshev et al., 2001). As such, the ncRNA derived from *BCYRN1* (BC200) shares strong sequence similarity with 7SL and Alu but includes more recently evolved, divergent sequence and structure features in its 3’-end (Tiedge et al., 1993). Like BC200, small NF90-associated RNA (snaR), first characterized in complex with RNA-binding protein NF90 (Figure 1; Parrott & Mathews, 2007), evolved from a monomeric Alu element that underwent successive deletion and expansion events (Parrott & Mathews, 2009). Like 7SL, Alu, BC200 and snaR are thought to influence translation and are commonly implicated in cancer. Here we explore the individual features of 7SL and its evolutionary derivatives, Alu, BC200, and snaR ncRNA.

### 5.1 | 7SL RNA: The signal recognition particle scaffold and riborepressor of p53 translation

**FIGURE 5:**
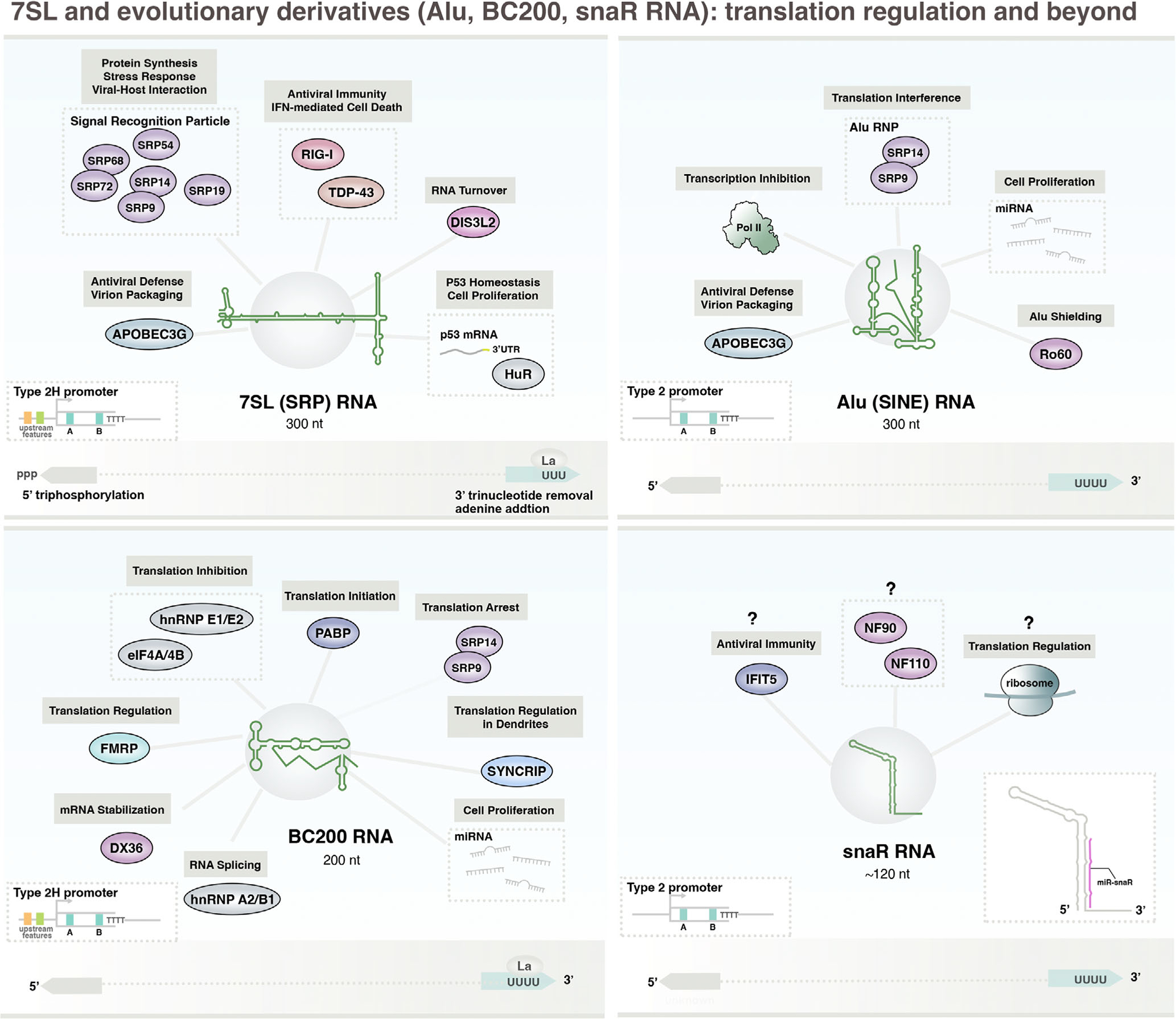
Basic features and functions of Pol III-transcribed 7SL RNA and its primate-specific evolutionary derivatives: Alu, BC200, and snaR ncRNA. Transcription of the Signal Recognition Particle (SRP) RNA, 7SL, is dependent on both gene internal and upstream regulatory sequences (type 2-hybrid promoter). Repetitive Alu SINE elements, evolutionarily derived from 7SL, possess only gene internal sequences, whereas subsequently evolved BCYRN1 (BC200 ncRNA) and snaR genes feature type 2-hybrid and type 2 promoters, respectively. Interactions with SRP proteins mediate translational arrest, whereas interactions with other RNA-binding proteins may intersect translation regulation through alternative mechanisms. Reported noncanonical functions include modulation of p53 homeostasis (7SL), transcription inhibition (Alu), miRNA sequestration (Alu, BC200), and noncanonical miRNA-biogenesis (snaR). High levels of 5’-PPP 7SL and Alu ncRNA molecules can promote RIG-I activation, and binding of IFIT5 to snaR-A indicates a potentially similar function as an endogenous immunostimulatory molecule.

First recognized as a low molecular weight RNA component in oncornaviruses in the 1970s, 7SL ncRNA was subsequently identified as an endogenous human cytoplasmic small RNA in 1976 (Bishop et al., 1970; Zieve & Penman, 1976). 7SL RNA is encoded by a limited set of functional genes in humans and undergoes post-transcriptional processing via 3’-oligo(U) trinucleotide removal, followed by adenine addition, generating a mature RNA approximately 300 nucleotides in length (Sinha et al., 1998). Genes encoding 7SL feature both gene internal as well as upstream regulatory features, termed type 2-hybrid promoters (Figure 5; Ullu & Weiner, 1985). 7SL RNA functions as the scaffold for signal recognition particles (SRP) proteins, which altogether co-translationally recognize and deliver secretory proteins to the endoplasmic reticulum (ER) membrane for translocation and proper folding (Akopian et al., 2013). 7SL first localizes to the nucleolus for SRP assembly prior to downstream SRP function in the cytoplasm, where it regulates the translation of nearly 30% of the human proteome (Akopian et al., 2013; Jacobson & Pederson, 1998).

As described for RMRP, 7SL is bound by DIS3L2, and accumulation of 3’-uridylated 7SL in cells lacking DIS3L2 suggests that modified and potentially misfolded 7SL RNA is ordinarily recognized and turned over by cytoplasmic DIS3L2-mediated decay (Pirouz et al., 2016). 7SL strongly retains 5’-triphosphorylation (5’-PPP) and, when unbound and unshielded by RNA-protein interactions, can induce immune signaling events through RIG-I detection (Nabet et al., 2017). Beyond interactions with SRP proteins, TDP-43 binding also plays an important role in shielding both 7SL and Alu RNA from RIG-I, preventing endogenous 7SL and Alu from triggering lethal RLR-dependent interferon responses (Dunker et al., 2021). However, 7SL RNA is also efficiently packaged into extracellular vesicles and can go on to function as exogenous immunostimulatory RNA through exosomal transfer (Nabet et al., 2017). In a similar nature, 7SL is selectively packaged into retroviral virions and can establish RNP complexes with viral proteins. Interactions between 7SL and APOBEC3G, a cytidine deaminase, can promote the recruitment of APOBEC3G and co-packaging in virions, facilitating APOBEC3G-mediated antiviral activities (Bach et al., 2008).

Upregulation of 7SL RNA is observed in multiple cancer contexts and, like Pol III-transcribed 5S rRNA and RMRP, 7SL also promotes cell proliferation through modulation of p53 homeostasis (Abdelmohsen et al., 2014). Specifically, RNA–RNA interactions between 7SL and the 3’-UTR of p53 mRNA competitively interfere with the binding of HuR, an RNA-binding protein that otherwise enhances p53 mRNA translation. Consistent with these observations, modulation of 7SL by disruption of FOXP3, a suppressor of 7SL transcription, induces upregulation of p53 and inhibition of tumor growth (Yang et al., 2016). The multifaceted intersection between 5S rRNA, RMRP, and 7SL with p53 homeostasis suggests Pol III transcription actively opposes p53 and downstream function. However, the pro-growth and immunestimulatory 7SL RNA may hold promise when used in the right context. For example, engineered CAR-T cells expressing 7SL can preferentially deliver 7SL to human immune cells, increasing cell expansion and reducing T cell exhaustion (Johnson et al., 2021). This strategy may be used in the future to exploit 7SL RNA as a novel delivery mechanism to improve immune response function and overcome immune-resistant tumors.

### 5.2 | Alu RNA: Repeat-derived ncRNA capable of modulating transcription and translation

Alu repeats represent the most abundant subtype of short interspersed nuclear elements (SINEs), a non-autonomous class of retrotransposons constituting over 10% of the human genome (Lander et al., 2001). First described as a family of 3’0-nucleotide DNA repeats digestible by restriction enzyme *AluI* (Houck et al., 1979), Alu elements were subsequently understood to be a dimeric fusion of ancestral 7SL Alu monomers, possessing A-box and B-box type 2 promoter elements (Figure 5; Quentin, 1992). Alu elements have further evolved into multiple subfamilies of varying sequence divergence and age, with the oldest Alu repeats (AluJ) most similar to the ancestral 7SL gene (Jurka & Smith, 1988). Alu elements are also present within Pol II-transcribed genes, where embedded Alu repeats are involved in diverse functions, including cryptic alternative splicing and the biogenesis of circRNA (Wilusz, 2015).

While most Alu elements are epigenetically repressed by histone H3K9 trimethylation (Varshney et al., 2015), a limited number are found to play important roles in chromatin organization and gene regulation (Crepaldi et al., 2013; Policarpi et al., 2017). With Pol III-transcribed Alu elements, the Alu ncRNA generated can go on to modulate both transcription and translation. In response to heat shock, Alu ncRNA binds RNA polymerase II at most promoters and inhibits transcription (Mariner et al., 2008). Alu ncRNA also reportedly represses gene expression through mRNA hybridization and biogenesis of Alu-derived miRNAs with similar functionality (Chen & Yang, 2017). With structural features analogous to the Alu domain of 7SL, Alu ncRNA is similarly capable of binding SRP proteins SRP9/14, assembling an Alu RNP complex that is also competent in ribosome stalling and translational interference (Ivanova et al., 2015). APOBEC3G, the 7SL-interacting antiviral restriction factor, interacts with and sequesters Alu ncRNA to inhibit L1-dependent retrotransposition (Chiu et al., 2006) and, in a similar fashion, the Y RNA-binding protein Ro60 (discussed below) interacts with Alu and prevents inflammatory gene expression patterns in human lymphoblastoid cells (Hung et al., 2015). Meanwhile, DUSP11, an RNA phosphatase and regulator of cellular 5’-triphosphate RNA, actively limits the accumulation of 5’-PPP Alu, which may otherwise activate RIG-I and downstream IFN responses (Burke et al., 2016).

In contrast to the inhibitory activities described for Alu RNA and the Alu RNP, overexpression of Alu ncRNA in cancer and other disease states leads to changes in transcription, translation, and cell proliferation in varying ways. Ectopic expression of specific Alu sequences in IMR90 cells reprograms the expression of cell cycle genes and promotes transition from G1 to S phase (Cantarella et al., 2019). This consequence was not observed in already transformed HeLa cancer cells, suggesting Alu ncRNA-driven effects are context-dependent. Consistent with this report, Alu expression did not affect proliferation in colorectal cancer cells, but otherwise induced epithelial-to-mesenchymal transition and cancer progression (Di Ruocco et al., 2018). Loss of DICER1, which processes and degrades Alu ncRNA, causes accumulation of Alu RNA and loss of miR-566, suggesting Alu may function as a molecular sponge as described for other Pol III-derived ncRNA (Di Ruocco et al., 2018). Accumulation of Alu RNA in retinal pigment epithelial cells, on the other hand, causes cytotoxicity (Kaneko et al., 2011), in line with reports of decreased viability in MCF7 breast cancer cells transfected with Alu and 7SL RNA (Baryakin et al., 2013). While much remains to be learned about the precise role of Pol III-derived Alu ncRNA, the demonstration of Alu-driven cell cycle progression and sustained proliferation in primary IMR90 implicates Alu as a potentially important factor in the early stages of cancer development (Cantarella et al., 2019).

### 5.3 | BC200 RNA: Primate-specific cytoplasmic RNA and multifunctional translational riboregulator

First discovered as a 200-nucleotide cytoplasmic RNA in monkey and human brain tissues (Watson & Sutcliffe, 1987), BC200 RNA shares strong sequence similarity with 7SL and Alu in its 5’-end and includes additional A-rich and C-rich domains in its central and 3’-end, respectively (Tiedge et al., 1993). BC200 ncRNA is encoded by a single copy gene, BCYRN1, which recruits Pol III through a type 2-hybrid promoter architecture possessing both gene internal and external regulatory features (Figure 5; Kim et al., 2017). BC200 ncRNA is selectively expressed in a subset of neurons and bound and stabilized by chaperone protein La through its 3’-oligo(U) tract (Kremerskothen, Nettermann, et al., 1998). With sequence and structural homology to 7SL and Alu ncRNA, BC200 also interacts with SRP proteins SRP9/14 and is similarly thought to induce translational elongation arrest (Kremerskothen, Zopf, et al., 1998). Through its unique 3’ C-rich domain, BC200 establishes interactions with hnRNP E1 and E2, as well as RHAU/DXX36, an RNA helicase thought to direct BC200 ncRNA to RNA species containing unwound quadruplex structures (Booy et al., 2016; Jang et al., 2017). BC200 is bound by several additional factors involved in translation regulation, including poly(A)-binding protein (PABP), eukaryotic initiation factor 4A (eIF4A), and RNA-binding proteins SYNCRIP and FMRP, which are thought to facilitate BC200-mediated local translational repression in neuronal dendrites (Sosinska et al., 2015).

BC200 is upregulated in cancer and shown to promote cell proliferation, invasion, and migration through multiple mechanisms. In MCF-7 cells, overexpression of BC200 leads to increased global translation and proliferation, whereas targeted downregulation decreases translation and cell growth, contradicting a generalized role for BC200 in translation repression (Booy et al., 2021; Singh et al., 2016). Knockout of BC200 also induces expression of a pro-apoptotic isoform of Bcl (Shin et al., 2017), and in both MCF-7 and HeLa cells, loss of BC200 decreases mRNA stability of S100A11, a factor important for cell motility (Singh et al., 2016), linking BC200 to alternative splicing regulation and RNA stabilization. In HeLa, BC200 also enhances migration by targeting a known tumor-suppressor miRNA, miR-138 (Peng et al., 2018). Molecular sequestration of other miRNAs is similarly reported in glioma, prostate, colorectal, and non-small-cell lung cancers, where BC200-mediated proliferation is associated with decreases in miR-125a-5p, miR-939–3p, miR-204–3p, and miR-149, respectively (Huo et al., 2020; Lang et al., 2020; Yang et al., 2020; Yu & Chen, 2019). BC200 is an unfavorable prognostic factor in these and other cancer contexts, such as bladder cancer, where exosomal transfer of BC200 RNA reportedly enhances VEGF-C/VEGFR3 signaling and lymphatic metastasis (Zheng et al., 2021). These and other BCYNR1-related studies altogether indicate that BC200 likely contributes to cancer progression through multiple mechanisms and may represent a promising therapeutic target for future research.

### 5.4 | snaR ncRNA: Recently evolved testis-specific ncRNA re-emergent in cancer

Small NF90-associated RNA (snaR) was first identified as a novel ncRNA upregulated in Epstein–Barr virus (EBV)-infected human B cells (Mrázek et al., 2007), and shortly after as the dominant RNA species bound by NF90, an isoform of interleukin enhancer binding factor 3 (ILF3), through in vivo cross-linking experiments (Parrott & Mathews, 2007). Genes encoding snaR RNA, which feature gene-internal A- and B-box (type 2) promoter elements (Figure 5), are evolutionarily restricted to higher order primates and, like BC200, evolved from a monomeric Alu element that underwent successive deletion and expansion events, generating multiple subsets of snaR genes with similar but divergent sequence features (Parrott & Mathews, 2009). snaR is also selectively expressed, with a specific snaR isoform, snaR-A, highly expressed in testis and also re-emergent in human cancers (Parrott et al., 2011). snaR-A RNA likely adopts an extended stem-loop secondary structure, with conserved apical stem-loop structures in most snaR subtypes. Though the post-transcriptional processing events of primary snaR ncRNA remain largely unexplored, evidence for snaR-A 3’-oligouridylation was identified in sequencing data following IFIT5 pulldown, which was enriched for snaR-A (Katibah et al., 2014). IFIT5 is a cytoplasmic, interferon-induced tetratricopeptide repeat (IFIT) family protein that co-localizes with RIG-I (Katibah et al., 2013) and modulates NF-κB signaling (Zhang et al., 2013), suggesting snaR-A may intersect and potentially transform IFIT5 preference for RIG-I and NF-κB signaling events. While little is known about snaR-A protein interactions beyond NF90 and IFIT5, such as whether snaR-A interacts with SRP proteins and other factors bound by 7SL family ncRNA, initial studies reported direct interactions with ribosomes and thus, like 7SL, Alu, and BC200, a putative role in translation regulation. Nevertheless, the primary function of snaR-A in testis or cancer contexts remains undefined.

Despite the comparatively limited understanding of snaR RNA interactions and function, studies examining the expression of snaR-A and its effects on cancer proliferation uncover a common pattern. In patients with ovarian cancer, snaR RNA is significantly upregulated and positively associated with the expression of GAB2, a signaling protein involved in transducing growth factors and cytokine responses (Huang et al., 2018). snaR-A is also upregulated in patients with hepatocellular carcinoma and, in HepG2 cells, overexpression of snaR-A upregulates TGF-β1 and promotes cell migration and invasion (Shi et al., 2019). Studies of snaR-A in other cell lines, including MDA-MD-231 and SK-BR3 (breast cancer), SPC-A1 and A549 (non-small cell lung cancer), and THP-1 (acute myeloid leukemia), identify snaR-A ncRNA as an important driver of cell proliferation and invasion (Ameli Mojarad & Pourmahdian, 2021; Lee et al., 2017; Lee, Jung, et al., 2016; van Bortle et al., 2022). While the mechanisms of snaR-A-mediated proliferation require further investigation, a noncanonical miRNA derived from snaR-A (miR-snaR) was recently discovered and shown to target the 3’-UTR of NME1, an inhibitor of metastasis (Stribling et al., 2021). Ectopic expression of miR-snaR, which is otherwise endogenously derived from Dicer-dependent processing of full-length snaR-A, promotes cell migration through repression of NME1 in MCF-7 cells (Stribling et al., 2021). Ectopic miR-snaR did not influence cell proliferation, suggesting snaR-A upregulation in cancer may drive growth and migration through multiple and potentially parallel mechanisms.

## 6 | Y RNA: MULTIFUNCTIONAL STEM BULGE SMALL RNA COMPONENTS OF THE RO60 RNP

**FIGURE 6:**
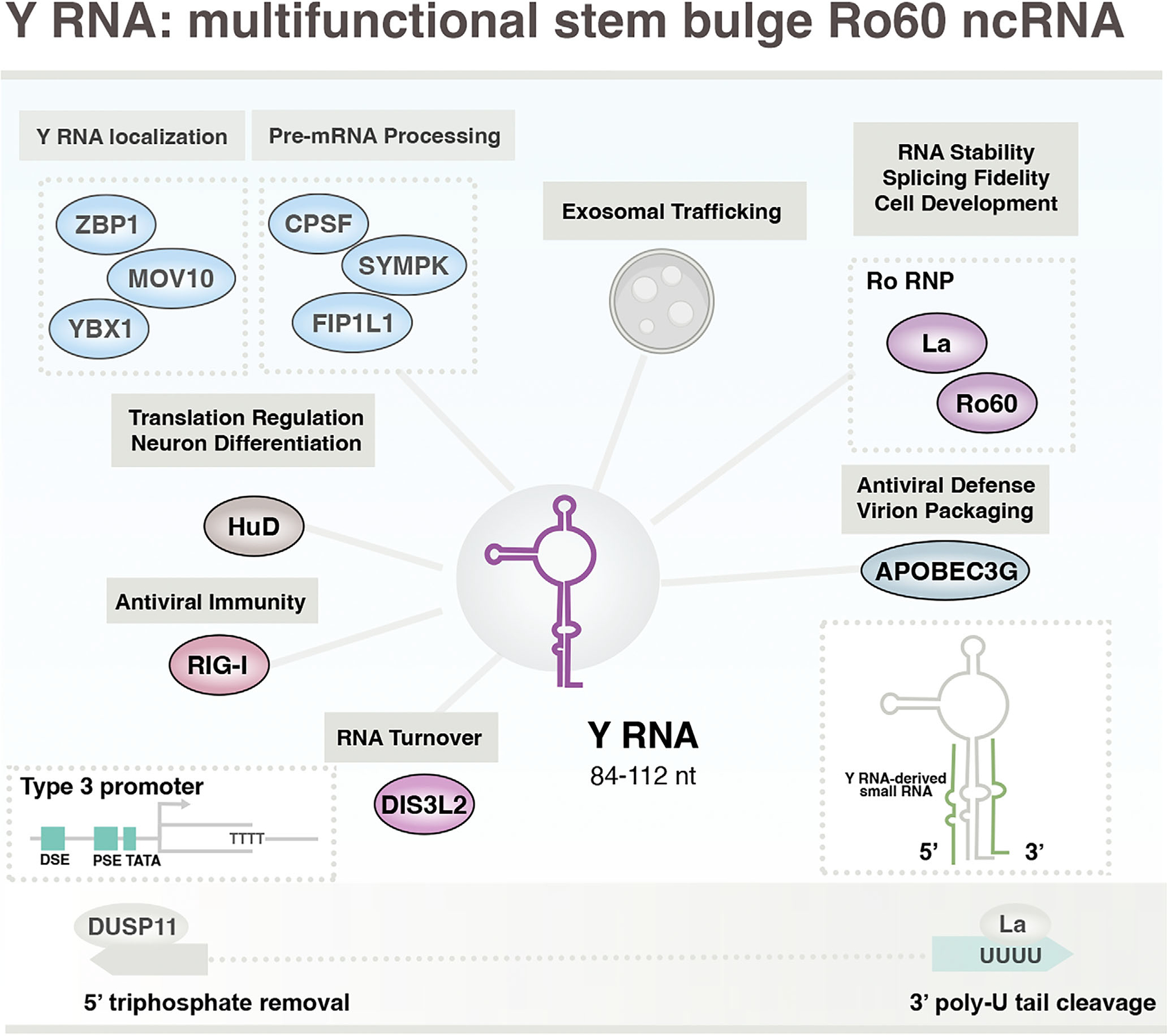
Pol III-transcribed Y RNAs are multifunctional stem bulge ncRNA species that modulate Ro60 function among other processes. RNA polymerase III is recruited to Y RNA genes via type 3 promoter elements. Y RNAs were first identified as components of the Ro60 RNP and implicated in RNA stability, but several additional roles are reported. Interactions with specific RNA-binding proteins regulate translation, including the sequestration of translation factor HuD which functionally counteracts neuron differentiation. Y RNAs are notably enriched within exosomes and similarly packaged into virions. Dynamic levels of endogenous 5’-PPP Y RNA molecules, which can increase in response to viral infection, activate RIG-I immune signaling pathways.

Y RNA was first discovered as an RNA component of Ro60 RNPs immunopurified by autoantibodies derived from patients with a form of systemic lupus (Figure 1; Lerner et al., 1981). RNA-binding protein Ro60 is implicated in RNA quality control pathways for binding misfolded and aberrant ncRNA, and interactions with Y RNA are thought to influence the ability of Ro60 to target specific RNA species (Sim & Wolin, 2011). Knockout of Y RNA in mammals also results in reduced levels of Ro60 and changes in the subcellular localization and protein network of Ro60, identifying Y RNAs as important regulators of Ro60 function (Leng et al., 2020). Y RNAs are evolutionarily conserved across vertebrate species, and similar stem-bulge ncRNA species with homologous structures are present in other species (Sim & Wolin, 2018). In humans, four distinct Y RNA species, ranging between 84 and 112 nucleotides, are generated by Pol III transcription at single-copy genes bearing type 3 promoter elements (Figure 6; Wolin & Steitz, 1983). Each Y RNA features a similar secondary structure, including a conserved lower stem domain important for Ro60 interactions, a stem bulge, a conserved upper stem domain implicated in DNA replication initiation, and more divergent apical loop domains which feature secondary stem loops involved in additional protein interactions (Kowalski & Krude, 2015). The chaperone protein La binds to the Y RNA 3’-oligo(U) tract and influences Y RNA stability, subcellular localization, and protein interactions (Simons et al., 1996). Y RNA processing includes 3’-oligo(U) tail trimming and 5’-PPP removal by DUSP11 (Burke et al., 2016). Increased levels of immunostimulatory 5’-PPP Y RNA, driven by changes in DUSP11 abundance and activity, can activate RIG-I and RLR-mediated immune signaling (Vabret et al., 2022). Cytoplasmic Y RNA is also targeted by TUT-DIS3L2 surveillance and the DIS3L2-mediated decay pathway (Pirouz et al., 2016; Ustianenko et al., 2016).

The protein interactome of Y RNA includes numerous factors that altogether implicate Y RNA in a wide range of cellular processes beyond Ro60 RNP function. In the nucleus, Y RNA interacts with replication initiation proteins, likely in complex (Zhang et al., 2011), and may play an essential function in DNA replication in humans (Christov et al., 2006). In the cytoplasm, Y RNA interacts with neuronal ELAV-like (nELAVL) translation factors (Scheckel et al., 2016) where, for example, Y RNA hY3 interacts with and sequesters HuD, effectively limiting HuD-mediated translation enhancement in neurons (Tebaldi et al., 2018). Y RNA is also notable for its significant enrichment in extra-cellular vesicles (EVs) and is analogously packaged into retroviral virions (Nolte-’t Hoen et al., 2012). In both EVs and viruses, Y RNA is detected along with several Y RNA-interacting proteins, including Ro60, multiple proteins involved in mRNA processing and splicing, and as described for 7SL and Alu, APOBEC3G (Driedonks & Nolte-’t Hoen, 2018). Beyond interactions with APOBEC3G and detection of 5’-PPP by RIG-I, unbound Y RNA can activate specific toll-like receptors (TLR; Greidinger et al., 2007), and thus exosomal transfer of Y RNA is likely to modulate innate immune responses through multiple pathways. In addition, Y RNA-derived small RNA (ysRNA) fragments, generated independent of the classical miRNA pathway (Nicolas et al., 2012), are also enriched within EVs and have been shown to activate caspase 3 and NF-κB signaling and are similarly capable of activating a specific toll-like receptor, TLR7 (Hizir et al., 2017).

As described for other Pol III-derived ncRNA, Y RNA is significantly overexpressed in multiple cancers, including solid tumors derived from bladder, cervix, colon, kidney, lung, and prostate cancers (Christov et al., 2008), and altered levels of ysRNA in related contexts suggests that modulation of both Y RNA expression and post-transcriptional processing events are common features during disease progression. In many cases, specific subsets of Y RNA and/or ysRNA are altered, suggesting context-specific mechanisms may underlie distinct patterns and downstream effects (Gulìa et al., 2020). Increased levels of Y RNA and ysRNA are also observed in extracellular compartments, including increased 3’-Y RNA fragments in blood serum from patients with breast cancer (Dhahbi et al., 2014) and increased hY4 in exosomes from patients with chronic lymphocytic leukemia (Haderk et al., 2017). hY5-associated small RNAs, mean-while, are enriched within EVs derived from K562 cells and can selectively induce apoptosis in non-cancerous primary cells, potentially enhancing microenvironment conditions for cancer progression (Chakrabortty et al., 2015). Notably, Ro60 and Ro60-dependent autoimmune conditions are also implicated in cancer progression (Bernatsky et al., 2013; Liu et al., 2018), yet more work is needed to better understand the contributions and potential interplay of Y RNA and Ro60 in tumorigenesis.

## 7 | VAULT RNA AND NC886: SMALL RIBOREGULATORS OF AUTOPHAGY, IMMUNE SIGNALING, AND OTHER PROCESSES

Vault RNAs (vtRNAs) were discovered as stably associated small RNA components of vault RNPs (Figure 1; Kedersha & Rome, 1986), massive 13 MDa assemblies implicated in a variety of processes but which continue to remain poorly understood (Kong et al., 1999). A related ncRNA, nc886, was first classified as a miRNA precursor and subsequently described instead as a novel vault RNA upregulated by EBV infection (Mrázek et al., 2007). Nc886 structurally resembles vtRNA, possesses similar gene regulatory features, and co-sediments with intact vault particles (Nandy et al., 2009). However, despite similar features and its initially described association with the vault complex, nc886 is now classified as neither miRNA nor vault RNA, but rather a 102-nucleotide cytoplasmic small RNA that functionally inhibits PKR, an interferon-induced kinase involved in innate immune pathways (Jeon et al., 2012). Based on historical context and structural similarity, we present a shared overview of vtRNA and nc886 features, functions, and reported connections to cancer.

### 7.1 | Vault RNA: RNA Component of cytoplasmic vault RNPs and riboregulators of autophagy

**FIGURE 7:**
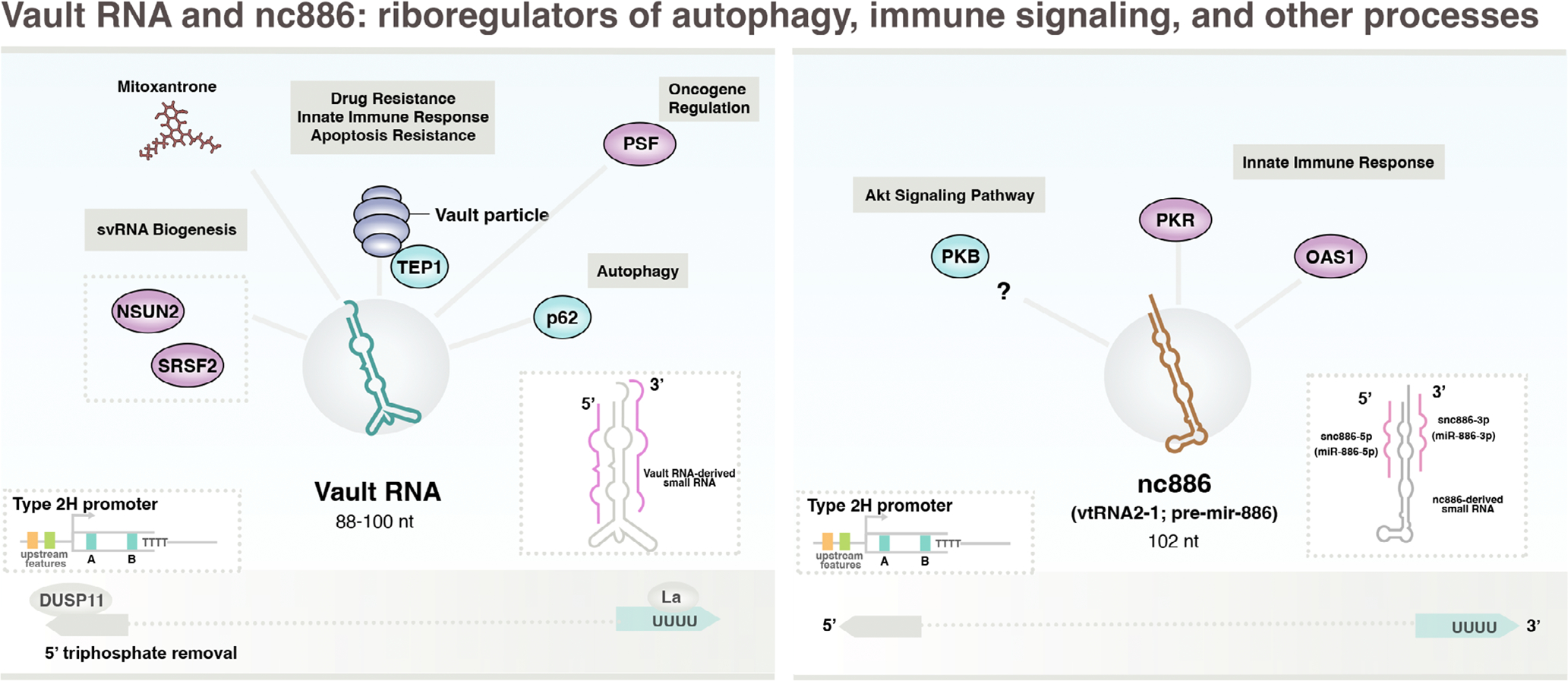
An overview of Pol III-transcribed vault RNA and nc886, structurally related riboregulators of autophagy and immune signaling, respectively. Both vault RNA genes and nc886 recruit RNA polymerase III via type 2-hybrid promoter sequences. Vault RNAs are named for their identified role in vault particles, though most vtRNAs function in other processes, including modulation of p62-mediated autophagy. Despite initial characterizations as a vault-associated RNA (vtRNA2–1), nc886 is not a component of vault particles and instead modulates the activity of Protein Kinase R (PKR), an interferon-induced serine/threonine kinase and dsRNA-binding protein. Both vtRNA and nc886 are further processed into smaller secondary ncRNAs with additional roles, including miRNA function.

In humans, vault RNA is encoded by a triple repeat of Pol III-transcribed genes, *VTRNA1*-*1*, *VTRNA1*-*2*, and *VTRNA1*-*3*, located on chromosome 5. Vault RNA genes possess type 2-hybrid promoter architectures that contain gene internal as well as additional upstream regulatory and response elements (Figure 7; van Zon et al., 2001). *VTRNA1*-*1* is the most highly expressed vtRNA gene, potentially as a result of a unique secondary B-box (B2) element located downstream (van Zon et al., 2001). vtRNAs range between 88 and 100 nucleotides in length and form extended stem-loop secondary structures defined by conserved stem domain sequences intervened by an internal, variable loop sequence (Stadler et al., 2009). Nascent vtRNA 5’-PPP is likely processed by DUSP11 (Burke et al., 2016), and chaperone protein La binds the 3’-oligo(U) tail of vtRNA, forming a stable RNP complex (Kickhoefer et al., 2002). Processing of vtRNA also includes NSUN2-mediated m^5^C methylation, which enhances the biogenesis of secondary vtRNA-associated small RNAs (vtsRNAs; Hussain et al., 2013). The binding of spliceosomal protein SRSF2 to vtRNA1–1 can restrict the processing of a specific vtsRNA, svRNA4 (Sajini et al., 2019), which like miR-snaR, functions as a noncanonical miRNA (Hussain et al., 2013).

vtRNA-associated vault particles are cytoplasmic assemblies comprised of 78 copies of major vault protein (MVP) and additional proteins, TEP1 and VPARP. The vault RNP features a massive barrel-like structure that is capable of rapid redistribution in response to external stimuli and is hypothesized to facilitate protein exchange between the cyto-plasm and nucleus (Frascotti et al., 2021). TEP1 binds to vtRNA and mediates the stable association of vtRNA within vault particles, though the functional significance of vault-associated vtRNA remains poorly understood (Kickhoefer et al., 2001). Moreover, it must be emphasized that most cellular “vault” RNA does not localize to vault particles (Kickhoefer et al., 2002), and recent characterization of vtRNA has implicated these ncRNA is many processes unrelated to vault RNPs. For example, vtRNA1–1 was recently shown to inhibit p62-mediated autophagy through direct interactions that preclude p62 oligomerization and downstream function (Horos et al., 2019). vtRNA1–1 also interacts with a DNA–RNA-binding protein, PSF, and competitively inhibits its promoter localization at specific oncogenes, releasing PSF-mediated gene repression and upregulating transcription (Chen et al., 2018). Transcriptome profiling in knockout cells confirm significant differential expression patterns in HeLa cells lacking vtRNA1–1, including upregulation of genes involved in PI3K/Akt and MAPK signaling pathways during serum starvation (Bracher et al., 2020). Knockout of vtRNA1–1 in HeLa increased the level of apoptosis under starvation conditions, whereas ectopic overexpression of vtRNA1–1 was previously shown to reduce apoptosis in human B cells (Amort et al., 2015), supporting a role for vtRNA1–1 in apoptosis resistance and cancer cell survival.

In addition to the pro-survival role of vtRNA1–1, overexpression and knockout experiments demonstrate that vtRNA1–1 promotes cancer cell proliferation as described for most of the Pol III transcriptome (Bracher et al., 2020; Chen et al., 2018; Ferro et al., 2022). A unique feature of vtRNA, however, is a purported role in cancer drug resistance, including evidence of direct binding to anti-cancer drugs. vtRNA1–1 and vtRNA1–2 bind mitoxantrone and decrease the efficacy of the chemotherapeutic compound in glioblastoma, leukemia, and osteosarcoma-derived cell lines (Gopinath et al., 2005; Gopinath et al., 2010). Multidrug-resistant cancer cell lines express elevated levels of vtRNA and higher levels of vault particles, which are also implicated in drug resistance (Kickhoefer et al., 1998). Knockout of vtRNA1–1 in hepatocellular carcinoma cells, meanwhile, increases the cytotoxicity of another chemotherapeutic agent, Sorafenib (Ferro et al., 2022). Though the precise mechanism of vtRNA-mediated drug resistance remains poorly understood and may be multifaceted, vtRNA represents a potentially promising biomarker and molecular target for future research.

### 7.2 | nc886 (vtRNA2–1): Cytoplasmic repressor of RNA-dependent protein kinase PKR

nc886 was initially classified as a pre-miRNA (pre-mir-886) based on the capture of small, putative miRNA species, miR-886–5p and miR-886–3p, in sequencing experiments. Following identification instead as a novel vtRNA-like ncRNA (Mrázek et al., 2007; Nandy et al., 2009; Stadler et al., 2009), a revised name, vtRNA2–1, was assigned and continues to remain in use today. However, subsequent studies failed to confirm the association of nc886 in vault particles, to which vtRNA1–1 is stably associated (Lee et al., 2011). Moreover, the gene encoding nc886, which also relies on a type 2-hybrid promoter architecture (Lee, 2022), diverges in sequence similarity in upstream elements that are otherwise shared by *VTRNA1–1, VTRNA1–2*, and *VTRNA1–3* (Figure 7; Stadler et al., 2009). Subsequent studies have instead identified distinct features and functions of nc886, suggesting *VTRNA2–1* requires re-classification and recognition as a distinct Pol III-transcribed gene subclass. Though the processing events of nc886 remain largely unmapped, we can expect that 5’-PPP removal by DUSP11 and nascent 3’-oligo(U) binding by chaperone La are likely events. nc886 RNA is also enriched in NSUN2 IP experiments (Khoddami & Cairns, 2013), suggesting m^5^C methylation may play a role in nc886 stability and processing as described for other Pol III-transcribed genes.

Protein Kinase R (PKR), an interferon-induced serine/threonine kinase and dsRNA-binding protein, binds nc886 with high affinity (Jeon et al., 2012; Lee et al., 2011). nc886 uniquely inhibits PKR activation, in contrast to the stimulatory effect of most dsRNA, and loss of nc886 results in apoptosis through PKR activation (Lee, Hong, et al., 2016). In vitro biochemical studies suggest nc886 can adopt two nonequivalent structures, differing in apical stem-loop structure involved in PKR repression, that either potently inhibits or weakly activates PKR, suggesting a more complex interplay between nc886 and PKR function (Calderon & Conn, 2017). The apical stem-loop of nc886 also activates OAS1, a cytosolic dsRNA sensor and activator of RNase L, in a conformer-specific manner, further supporting a central role for nc886 in modulating innate immune response to viral infection and other processes (Calderon & Conn, 2018).

In contradiction to the pattern that emerges for most of the Pol III transcriptome, downregulation rather than overexpression of nc886 is observed in multiple cancer contexts and associated with poor survival outcomes. Promoter methylation, which drives epigenetic silencing of nc886, increases in AML (Treppendahl et al., 2012), esophageal squamous cell carcinoma (ESCC; Lee, Lee, et al., 2014), gastric cancer (Lee, Park, et al., 2014), cholangiocarcinoma (Kunkeaw et al., 2013), prostate cancer (Fort et al., 2018), and breast cancer contexts (Park et al., 2017). Described as a putative tumor suppressor, nc886 overexpression decreases cell proliferation and invasion (Fort et al., 2018) and mechanistically suppresses the induction of specific oncogenes (Lee, Lee, et al., 2014) as well as the pro-survival AKT pathway (Im et al., 2020). Evidence suggests secondary small RNAs derived from nc886 may also contribute to tumor suppressor function through miRNA activity (Fort et al., 2020). However, it must be noted that both nc886 and nc886-derived miRNAs are conversely associated with cancer progression in ovarian and cervical cancers, suggesting that the reported nc886-mediated tumor suppressor function is context-specific (Ahn et al., 2018; Kong et al., 2015).

## 8 | AN EXPANDED VIEW OF THE POL III TRANSCRIPTOME

The Pol III transcriptome extends beyond the genes and small noncoding RNA species highlighted in this review. In addition to Alu elements, mammalian-wide interspersed repeat (MIR) elements represent a highly abundant and ancient SINE subclass in humans (Jurka et al., 1995). MIR repeats are tRNA-derived SINEs shown, like Alu, to function as enhancers as well as putative insulator regulatory elements (Jjingo et al., 2014; Wang, Vicente-García, et al., 2015). However, the level and function of Pol III-derived MIR ncRNA remain poorly defined. Pol III transcription at MIR repeats has also been shown to directly influence the expression of overlapping Pol II-transcribed genes through transcriptional interference (Gerber et al., 2020; Yeganeh et al., 2017). More broadly, acute depletion of Pol III perturbs Pol II transcription by impairing the recruitment of the FACT complex, suggesting cross-talk between Pol III and Pol II activity extends to numerous protein-coding genes sensitive to local chromatin architecture (Jiang et al., 2022). Evidence suggests Pol III may also be capable of transcription at A/T-rich Pol II promoters, indicating a more direct and potentially competitive intersection between Pol III and Pol II than currently appreciated (Duttke, 2014).

The boundaries of Pol III activity extend also into the domain of viral-host interactions. It is well established that Pol III transcription, which is upregulated by Epstein–Barr Virus (EBV)-encoded nuclear antigen 1 (EBNA1), is responsible for the expression of EBV-encoded RNAs (EBERs; Felton-Edkins et al., 2006; Owen et al., 2010). Pol III is unique among the RNA polymerase machineries for its subcellular distribution in both nuclear and cytoplasmic subcompartments. Cytosolic Pol III is capable of synthesizing 5’-PPP RNA from A/T-rich templates and DNA viruses which, like specific endogenous Pol III ncRNAs, are detected by RIG-I and induce IFN responses (Chiu et al., 2009). Recent work has demonstrated that, like EBV, Pol III activity is also stimulated by Herpes Simplex Virus-1 (HSV-1) and in turn, binds the HSV-1 genome and mediates expression of specific HSV-1 encoded genes (Dremel et al., 2022). Both EBV and HSV-1 are estimated to be highly ubiquitous within human populations, yet the interplay of viral infection, Pol III activity, and cancer incidence and progression remain uncharted but important areas for future research.

## 9 | CONCLUSIONS

**FIGURE 8:**
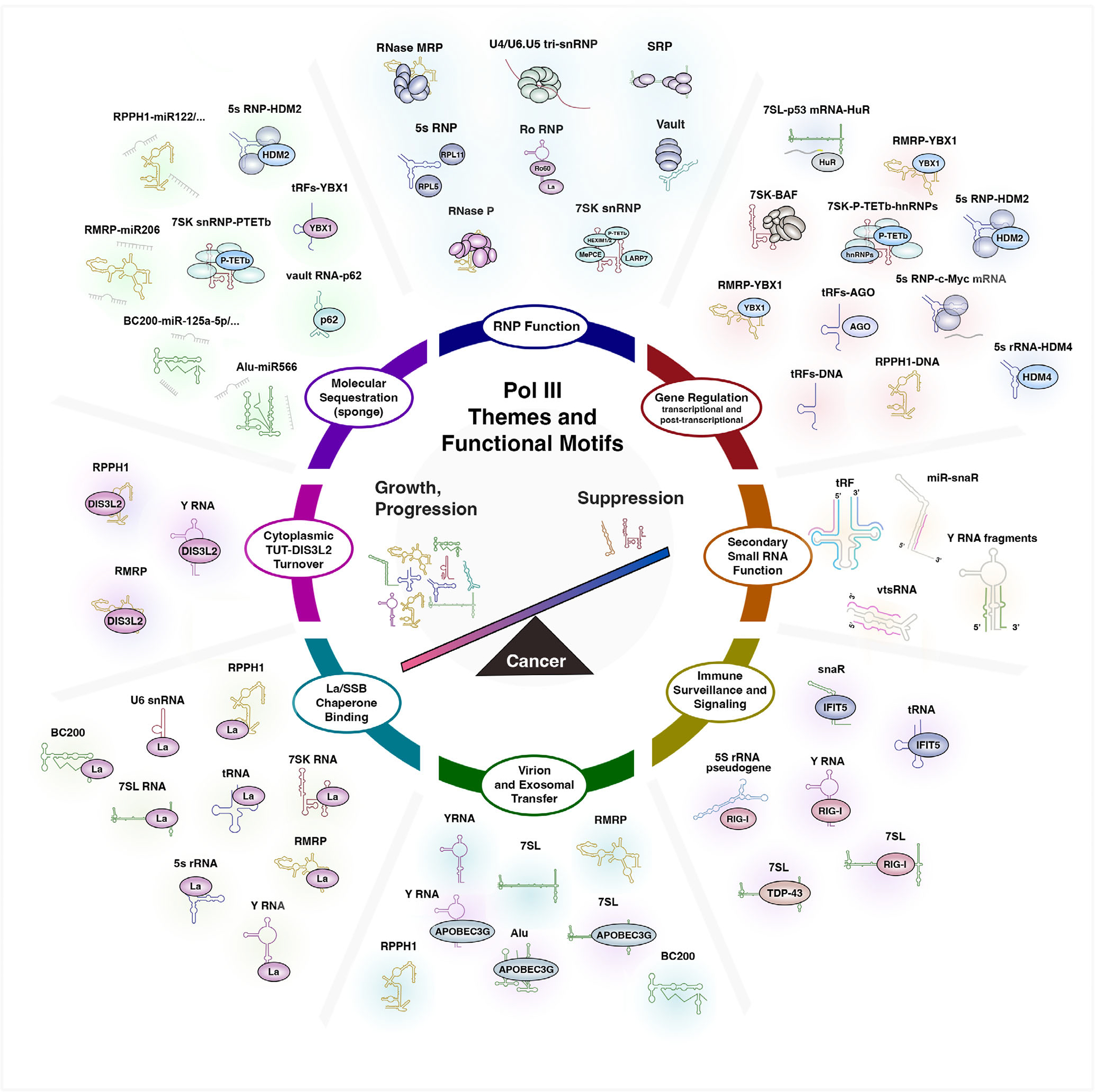
A generalized model of the Pol III transcriptome: emergent patterns and functional motifs. The illustration highlights general themes and specific examples, but is not exhaustive. Pol III-transcribed genes largely function through ribonucleoprotein complexes for which most ncRNA subclasses were first identified (Figure 1). However, numerous secondary functions are reported, including examples of transcriptional and post-transcriptional gene regulation and miRNA function by secondary small RNA fragments, as well as ncRNA-mediated sequestration of specific miRNAs and proteins. Uncapped and unshielded Pol III transcripts containing 5’-PPP can activate endogenous immune pathways and are also identified in the cargo of extracellular vesicles and viral particles with implications for extracellular function. The Pol III transcriptome is likely universally bound by the chaperone protein La/SSB, and cytoplasmic levels of multiple ncRNA subclasses are controlled through DIS3L2 surveillance pathways. In the context of cancer, the Pol III transcriptome appears to overwhelmingly favor growth and cancer progression, though 7SK snRNA and nc886 are identified as putative tumor suppressor RNAs.

The small noncoding RNAs derived from Pol III transcription are understood to play critical roles in basic and diverse cellular processes. In many cases, ncRNA species were historically identified as core components of the ribonucleoprotein complexes central to these processes (Figure 1). This review has sought to revisit the initial characterizations for each Pol III-derived ncRNA and to outline the features, protein interactions, and cancer connections reported for the Pol III transcriptome in humans (Figures 2–7). Upon examination of each RNA subtype, many noncanonical activities are noted. When taken together, recurrent patterns and themes emerge that collectively link Pol III transcription to activities far beyond the textbook models ascribed for each RNA (Figure 8).

Perhaps most apparent among the shared motifs is the molecular sequestration activities described for most ncRNAs. Though sequestration of PTEF-b is well established for 7SK, recent work has uncovered analogous functions for vault RNA (p62 and autophagy), 5S rRNA (HDM2 and p53 homeostasis), and tRNA (YBX1 and RNA stability). Like 7SK, nearly all Pol III-derived ncRNAs also modulate gene expression in some form, including examples of direct transcription regulation (RPPH1/RNaseP), altered mRNA stability (RMRP and YBX1), and the biogenesis of secondary small RNA fragments with reported miRNA function (tRFs, miR-snaR, vtsRNA, etc.). In parallel to protein sequestration, many reports identify similar roles for the Pol III transcriptome in sequestering miRNAs (e.g., RMRP-miR-206), counteracting miRNA-mediated downregulation of genes typically involved in cell growth.

In the case of sequestration, questions remain as to what degree such interactions modulate the availability and/or functionality of the molecular sponge itself. In some cases, noncanonical ncRNA interactions may conceivably help to shield cytoplasmic and immunostimulatory signatures, such as detection of 5’-PPP and ensuing activation of RIG-I interferon responses (Figure 8). Shared protein interactions and processing events, such as chaperone protein La, TDP-43, DIS3L2, DUSP11, and NSUN2, raise additional questions about competition among the ncRNA subtypes for shared molecular partners. In such a case, knowledge of the underlying stoichiometry for all RNA and protein constituents may be necessary to fully understand context-specific patterns and functional activities reported for a given Pol III-derived ncRNA.

Collectively, the Pol III transcriptome is overwhelmingly systematized for supporting and promoting cell growth (Figure 8). While intuitive in the sense that most Pol III-derived ncRNAs support protein synthesis and RNA maturation in some form, the additional intersections of ncRNA and p53 homeostasis (5S rRNA, RMRP, 7SL), putative feed-back mechanisms that coordinate the Pol III transcriptome with transcription and post-transcriptional gene regulation (RPPH1, tRFs, RMRP, 7SL), the sequestration of specific miRNAs targeting drivers of growth (BC200, Alu, RMRP, RPPH1), and the extracellular circulation of immunomodulatory ncRNAs (Y RNA, 7SL, etc.) represent extended mechanisms through which Pol III transcription is likely to enhance proliferation as well as cancer migration and invasion. The collective role of the Pol III transcriptome in cancer progression is further supported by recent evidence that even transient inhibition of RNA polymerase III is sufficient to diminish tumor initiation in mouse models (Petrie et al., 2019).

Nevertheless, our generalized model of the Pol III transcriptome, which emphasizes recurrent patterns and roles of small ncRNA in cancer, is inherently oversimplified. Patterns of enhanced proliferation and cancer progression are undeniably complicated by counterexamples in which Pol III-derived ncRNAs play prominent roles in either suppressing transcription and translation or otherwise promoting cellular differentiation. Perhaps most notably, 7SK snRNA is described as a putative tumor suppressor and is primarily recognized for mediating transcriptional repression. Similarly, 7SL and 7SL-derived ncRNAs mediate translational repression though, in the case of 7SL, this intermediate step contributes to further protein synthesis. We highlight evidence that full-length vtRNA promotes cell survival and proliferation, yet dynamic regulation of vault-associated small RNA (vsRNA) contributes to efficient cellular differentiation (Sajini et al., 2019), indicating a nuanced, multi-modal contribution of Pol III transcription to cell state and function that is dependent on downstream factors. Like 7SK, nc886 primarily functions as an inhibitory molecule and has been implicated as a tumor suppressor in multiple cancers, though this too appears to be context-dependent. Identifying and further deconstructing the full breadth of activities linked to the Pol III transcriptome is therefore critical for fully understanding the mechanisms underlying cancer progression and discovering new and improved strategies for therapy.

## Data Availability

Data sharing is not applicable to this article as no new data were created or analyzed in this study.
